# Effect of Broad-Spectrum Antibiotic De-escalation on Critically Ill Patient Outcomes: A Retrospective Cohort Study

**DOI:** 10.1007/s44197-023-00124-1

**Published:** 2023-06-09

**Authors:** Namareq Aldardeer, Ismael Qushmaq, Bashayer AlShehail, Nadia Ismail, Abrar AlHameed, Nader Damfu, Mohammad Al Musawa, Renad Nadhreen, Bayader Kalkatawi, Bashaer Saber, Mohannad Nasser, Aiman Ramdan, Abrar Thabit, Mohammed Aldhaeefi, Abeer Al Shukairi

**Affiliations:** 1 Pharmaceutical Care Division, King Faisal Specialist Hospital and Research Center, MBC J-11, P.O. Box 40047, Jeddah, 21499 Saudi Arabia; 2 Section of Critical Care Medicine, Department of Medicine, King Faisal Specialist Hospital and Research Center, Jeddah, Saudi Arabia; 3 Pharmacy Practice Department, College of Pharmacy, Imam Abdulrahman Bin Faisal University, Dammam, Saudi Arabia; 4 Pharmaceutical Care Division, King Fahad Hospital of the University, Al-Khobar, Saudi Arabia; 5 Pharmaceutical Care Division, King Faisal Specialist Hospital and Research Center, Madinah, Saudi Arabia; 6 Infection Prevention and Control Department, King Abdul Aziz Medical City, Jeddah, Saudi Arabia; 7 Faculty of Medicine, Mansoura University, Mansoura, Egypt; 8 Department of Clinical and Administrative Pharmacy Sciences, College of Pharmacy, Howard University, Washington, DC USA; 9 Pharmacy Practice Department, Faculty of Pharmacy, King Abdulaziz University, Jeddah, Saudi Arabia; 10 Department of Medicine, King Faisal Specialist Hospital and Research Center, Jeddah, Saudi Arabia; 11 College of Medicine, Alfaisal University, Riyadh, Saudi Arabia

**Keywords:** Antibiotics, De-escalation, Stewardship, Broad spectrum, Superinfection

## Abstract

**Purpose:**

Antibiotic de-escalation (ADE) in critically ill patients is controversial. Previous studies mainly focused on mortality; however, data are lacking about superinfection. Therefore, we aimed to identify the impact of ADE versus continuation of therapy on superinfections rate and other outcomes in critically ill patients.

**Methods:**

This was a two-center retrospective cohort study of adults initiated on broad-spectrum antibiotics in the intensive care unit (ICU) for ≥ 48 h. The primary outcome was the superinfection rate. Secondary outcomes included 30-day infection recurrence, ICU and hospital length of stay, and mortality.

**Results:**

250 patients were included, 125 in each group (ADE group and continuation group). Broad spectrum antibiotic discontinuation occurred at a mean of 7.2 ± 5.2 days in the ADE arm vs. 10.3 ± 7.7 in the continuation arm (*P* value = 0.001)**.** Superinfection was numerically lower in the ADE group (6.4% vs. 10.4%; *P* = 0.254), but the difference was not significant. Additionally, the ADE group had shorter days to infection recurrence (*P* = 0.045) but a longer hospital stay (26 (14–46) vs. 21 (10–36) days; *P* = 0.016) and a longer ICU stay (14 (6–23) vs. 8 (4–16) days; *P* = 0.002).

**Conclusion:**

No significant differences were found in superinfection rates among ICU patients whose broad-spectrum antibiotics were de-escalated versus patients whose antibiotics were continued. Future research into the association between rapid diagnostics with antibiotic de-escalation in the setting of high resistance is warranted.

**Supplementary Information:**

The online version contains supplementary material available at 10.1007/s44197-023-00124-1.

## Introduction

Sepsis and septic shock are considered among the leading causes of death in hospitals, with mortality reaching up to 30% and 38%, respectively [1–3]. Thus, administering proper empiric antibiotic therapy in these patients is highly recommended to minimize the risk of suboptimal coverage and eventually reduce patient mortality [4]. Antibiotic selection is based on patient risk factors of developing methicillin-resistant *Staphylococcus aureus* (MRSA) and multidrug-resistant (MDR) infections [4]. Prolonged use of broad-spectrum antibiotics that covers these infections might lead to the development of bacterial resistance [5–7].

The Infection Section of the European Society of Intensive Care Medicine (ESICM) and the European Society of Clinical Microbiology and Infectious Diseases (ESCMID) consensus defined antibiotic de-escalation (ADE) in critically ill patients as the process of replacing a broad-spectrum antibiotic with an agent of a narrow-spectrum or lower ecological impact or stopping components of antibiotic combination therapy [8]. ADE is part of the antibiotic stewardship initiative that aims to reduce the unjustified prolonged broad-spectrum antibiotic use and eventually decrease emergent resistance and antibiotic-related side effects [9, 10]. ADE approach is recommended within 24 h of definitive culture results and assessed within 72 h of empiric therapy [8, 11]. Using biomarkers, mainly procalcitonin, with clinical evaluation can aid in the discontinuation of antibiotics and decrease the duration of therapy [12]. Nonetheless, procalcitonin use in ADE is still limited and needs further study [4, 8].

The international survival sepsis campaign guidelines recommended daily assessment of ADE over providing fixed durations of therapy. A meta-analysis conducted by this guideline in the ADE group showed a significant decrease in short-term mortality (RR, 0.72; 95% CI 0.57–0.91) and a reduction in hospital length of stay (MD − 5.56 days; 95% CI − 7.68 to − 3.44). However, the differences in hospital length of stay (more than 90 days) and ICU length of stay were not significant [4].

According to the DIANA study, a multicenter retrospective cohort study, the ADE proportion of empirical antibiotics in adult critically ill patients was only 16% [13]. On the other hand, based on a single-center study conducted in Saudi Arabia, the ADE proportion was reported to be 48% in intensive care unit (ICU) patients [14]. Moreover, Japanese populations from the DIANA study were further analyzed to study the impact of broad-spectrum antibiotics (given beyond 72 h) on the detection of new MDR bacteria. That resulted in a significantly higher detected bacteria in the broad-spectrum antibiotics group compared to the narrow-spectrum one (11.9% vs. 4.2%, *P* = 0.042) [15]. A randomized controlled study found that the median ICU length of stay was longer in the ADE group compared to the continuation group (9 days [interquartile range (IQR) 5–22] compared to 8 days [IQR 4–15]; *P* = 0.71). Reported superinfections were 27% in the ADE group and 11% in the continuation group (*P* = 0.03) [16].

Currently, limited data are available regarding the effect of ADE on superinfections rate and infection recurrence. Hence, our study aim was to identify the impact of ADE compared to the continuation of therapy on the rate of superinfections, as well as clinical and microbiological outcomes in ICU patients.

## Methods

### Study Settings

This retrospective cohort study was conducted in the ICUs of two tertiary care academic hospitals (King Faisal Specialist Hospital in Jeddah (KFSHRC-J) and King Fahad Hospital of the University (KFHU) in Alkhobar) in Saudi Arabia between January 1st, 2022 and March 31st, 2022. The centers have ICU capacities of 18 and 26 beds, respectively. Both centers have established stewardship programs. The participating centers obtained local institutional review board approval with a waiver for informed consent (reference numbers IRB 2021-65/ KFHU and 2022-11-276, respectively).

### Patients and Data Collection

All patients aged 18 years or older were eligible for enrollment if they were admitted to the ICU and were initiated on an empiric anti-pseudomonal broad-spectrum antibiotic (piperacillin/tazobactam, ceftazidime, cefepime, meropenem, imipenem/cilastatin, ceftazidime/avibactam, or ceftolozane/tazobactam) for at least 48 h [17, 18]. Patients who were on broad-spectrum antibiotics for more than 48 h before ICU admission and patients who died or had their antibiotics stopped within 48 h of ICU admission were excluded.

Collected data included patients’ demographics, comorbidities, illness severity score, use of mechanical ventilation, de-escalation status, and total antibiotic duration. Data on cultures, sites of the microbiologically confirmed infection, and isolated organisms were also collected. The incidence of superinfection, infection recurrence, mortality, and length of stay were collected for outcome measures.

### Study Endpoints

The primary endpoint was the rate of superinfection based on ADE status. Secondary endpoints included rate of 30-day infection recurrence, requiring ICU re-admission within 30 days of ICU discharge, ICU and hospital length of stay, ICU mortality, in-hospital mortality, and frequency of broad-spectrum antibiotic re-escalation.

### Definitions

ADE was defined as replacing a broad-spectrum antibiotic with an agent of a narrower spectrum of activity (supplementary appendix Fig. e1) after the start of broad-spectrum empirical treatment [8]. The discontinuation of at least one anti-pseudomonal broad-spectrum antibiotic agent or the discontinuation of MRSA coverage was not considered de-escalation in our study. Antibiotic re-escalation was defined as the resumption of a broad-spectrum treatment justified by a clinical worsening, not necessarily related to the initial infection [19, 20]. We did not consider a timeframe for re-escalation in our definition; however, any upgrade of the antibiotic based on the spectrum of activity (supplementary appendix Fig. e1) was considered a re-escalation. 30-day recurrence was defined as culture positive for the same organism isolated from index culture, counted 30 days from the end of treatment [21]. Superinfection was defined as the isolation of a pathogen responsible for a subsequent infection that required the initiation of therapy and a species different from the initially isolated pathogen [22].

### Statistical Analysis

Data were presented as frequencies and percentages for categorical data and mean and standard deviation (SD) or median and interquartile range (IQR) for continuous data. Chi-square or Fisher exact tests, as appropriate, were used to test significant differences between categorical variables. Student’s t-test or Mann–Whitney test, as appropriate, were used to test significant differences between continuous variables. All reported *P* values were two tailed. A *P* value < 0.05 was considered significant. SPSS (Version 25.0. Armonk, NY: IBM Corp) was used for all statistical analyses. To attain a power of 90%, a sample size of 250 patients (125 in each group) was needed based on an estimated difference of 16% in superinfection rate between the ADE and the continuation group and an α-error probability of 5%. The effect size was determined based on findings from a previous study, where the superinfection rate was 27% in the ADE group and 11% in the continuation group [16]. Data collection was stopped once we reached the calculated sample size.

## Results

Of 366 screened ICU patients, 250 were included in the current analysis, 125 in the ADE group, and 125 in the continuation group (supplementary appendix Fig. e2). De-escalation included those who had de-escalation without the need for re-escalation (*n* = 68, 27.2%) and those who had de-escalation then re-escalation (*n* = 57, 22.8%) (supplementary appendix Fig. e3).

Table 1 shows the demographic and clinical characteristics of the patients. The average age was 61.1 ± 19.7 years, with 48.4% of the patients above 65 years. The most documented ICU admission diagnoses were respiratory failure (37.2%), followed by sepsis or septic shock (22.4%). The majority (80.4%) of the patients had at least one comorbidity with an average Charlson comorbidity index score of 4.2 ± 2.9. Approximately 16% of the patients were positive for severe acute respiratory syndrome coronavirus 2 (SARS-CoV-2) at the time of ICU admission. Compared with those who did not have de-escalation, those who had de-escalation had a less respiratory failure (*P* = 0.006), number of comorbidities (*P* < 0.001), Charlson comorbidity index score (*P* = 0.003), localized and metastatic solid tumors (*P* = 0.026 and *P* = 0.003, respectively).

**Table 1:** Baseline characteristics and demographic of the study patients

Characteristic	De-escalation status		Total (*n* = 250)	*P* value
	Yes (*n* = 125)	No (*n* = 125)		
Age (years)	60.3 ± 21.0	61.8 ± 18.3	61.1 ± 19.7	0.537
Age groups (years)				
≤ 50	38 (30.4)	32 (25.6)	70 (28.0)	0.082
51–65	22 (17.6)	37 (29.6)	59 (23.6)	
> 65	65 (52.0)	56 (44.8)	121 (48.4)	
Weight (kg)	74.9 ± 20.2	78.6 ± 25.1	76.8 ± 22.8	0.201
Height (cm)	162.5 ± 13.6	160.6 ± 14.6	161.6 ± 14.1	0.289
Body mass index (kg/m^2^)	28.1 ± 7.2	29.4 ± 8.8	28.8 ± 8.0	0.185
BMI group				
< 30 kg/m^2^	81 (64.8)	73 (58.4)	154 (61.6)	0.298
≥ 30 kg/m^2^	44 (35.2)	52 (41.6)	96 (38.4)	
Days from hospital admission to ICU admission	3.2 ± 8.0	6.8 ± 24.6	5.0 ± 18.4	0.636
ICU admission diagnosis				
Respiratory failure	36 (28.8)	57 (45.6)	93 (37.2)	0.006
Sepsis/septic shock	24 (19.2)	32 (25.6)	56 (22.4)	0.225
Other shock	10 (8.0)	16 (12.8)	26 (10.4)	0.214
Post-surgery	14 (11.2)	7 (5.6)	21 (8.4)	0.110
Charlson comorbidity index	3.7 ± 2.7	4.8 ± 3.0	4.2 ± 2.9	0.003
ICU scores upon ICU admission				
APACHE score	19.5 ± 7.8	21.4 ± 8.8	20.5 ± 8.3	0.065
SOFA score	7.5 ± 3.4	7.7 ± 2.9	7.6 ± 3.1	0.796
Ventilation status at antibiotic initiation				
High flow nasal cannula	9 (7.2)	15 (12.0)	24 (9.6)	0.198
Noninvasive ventilation	12 (9.6)	16 (12.8)	28 (11.2)	0.422
Invasive ventilation	76 (60.8)	70 (56.0)	146 (58.4)	0.441
Others	32 (25.6)	29 (23.2)	61 (24.4)	0.659
Patient SARS-CoV-2 status at ICU admission				
Yes	16 (12.8)	24 (19.2)	40 (16.0)	

Data are presented as mean ± SD or *n* (%)

APACHE, Acute Physiology and Chronic Health Evaluation; CKD, chronic kidney disease; COPD, chronic obstructive pulmonary disease; SARS-CoV-2, severe acute respiratory syndrome coronavirus 2; CVA, cerebrovascular accident; ICU, intensive care unit; SOFA, Sequential Organ Failure Assessment; TIA, transient ischemic attack

The details of antibiotic therapy used in the study patients are listed in Table 2. The most frequently used primary broad-spectrum antibiotics were meropenem (54%), followed by piperacillin/tazobactam (30.8%) and ceftazidime–avibactam (7.2%). Primary broad-spectrum antibiotics were continued for 8.7 ± 6.7 days. Compared with those who did not have de-escalation, patients in the ADE group had fewer antibiotics used before ICU admission (*P* < 0.001), less meropenem use, and more piperacillin/tazobactam use as primary broad-spectrum antibiotics (*P* < 0.001 for each), shorter duration of use of primary broad-spectrum antibiotics (*P* < 0.001), and less use of concurrent antibiotics (*P* = 0.007). Table 3 shows the microbiologic findings, where the rate of microbiological culture positivity was 39.6% of all the patients, predominantly with Gram-negative organisms (28.8%). The most isolated organisms were *Klebsiella pneumoniae* (36.4%), *Escherichia coli* (23.2%), *Pseudomonas aeruginosa* (14.1%), *Coagulase-negative Staphylococci* (14.1%), *Staphylococcus aureus* (13.1%), and *Acinetobacter baumannii* (10.1%).

**Table 2:** Antibiotic use among included patients

	De-escalation status		Total (*n* = 250)	*P* value
	Yes (*n* = 125)	No (*n* = 125)		
Antibiotics used prior to ICU admission	30 (24.0)	74 (59.2)	104 (41.6)	< 0.001
Primary broad-spectrum antibiotics				
Meropenem	43 (34.4)	92 (73.6)	135 (54.0)	< 0.001
Piperacillin/tazobactam	67 (53.6)	10 (8.0)	77 (30.8)	< 0.001
Ceftazidime–avibactam	6 (4.8)	12 (9.6)	18 (7.2)	0.142
Cefepime	3 (2.4)	7 (5.6)	10 (4.0)	0.334
Imipenem/cilastatin	3 (2.4)	4 (3.2)	7 (2.8)	> 0.99
Ceftolozane/tazobactam	3 (2.4)	0 (0.0)	3 (1.2)	0.247
Days from ICU admission to broad-spectrum antibiotic initiation	0.0 (0.0–2.0)	0.0 (0.0–1.5)	0.0 (0.0–2.0)	0.676
Duration of use of primary broad-spectrum antibiotics (days)	7.2 ± 5.2	10.3 ± 7.7	8.7 ± 6.7	< 0.001
Discontinuation of broad-spectrum antibiotics in 48–72 h	32 (25.6%)	15 (12.0%)	47 (18.8%)	0.006
Concurrent use of antibiotics	41 (32.8)	62 (49.6)	103 (41.2)	0.007
Total duration of the antibiotic course^a^	12.4 ± 8.3	11.8 ± 8.5	12.1 ± 8.4	0.245

Data are presented as mean ± standard deviation (SD) or *n* (%); days to antibiotic initiation presented as median and interquartile range (IQR)

^a^Including the broad-spectrum and the de-escalated antibiotic but not including the duration of antibiotic re-escalation after de-escalation

**Table 3:** Microbiologic findings

	De-escalation status		Total (*n* = 250)	*P* value
	Yes (*n* = 125)	No (*n* = 125)		
History of MDR or XDR infections 12 months prior to ICU admission	14 (11.2)	15 (12.0)	29 (11.6)	0.843
Culture positive	37 (29.6)	62 (49.6)	99 (39.6)	0.001
Culture site^a^				
Blood	12 (9.6)	30 (24.0)	42 (16.8)	0.002
Tracheal aspirate	20 (16.0)	20 (16.0)	40 (16.0)	> 0.99
Urine	6 (4.8)	22 (17.6)	28 (11.2)	0.001
Wound	5 (4.0)	5 (4.0)	10 (4.0)	> 0.99
Surgical drain	2 (1.6)	0 (0.0)	2 (0.8)	0.498
Cerebrospinal fluid	0 (0.0)	1 (0.8)	1 (0.4)	> 0.99
Organism				
Enterobacterales	19 (51.4)	38 (61.3)	57 (57.6)	0.333
* Pseudomonas aeruginosa*	7 (18.9)	7 (11.3)	14 (14.1)	0.292
Coagulase-negative *Staphylococci*	1 (2.7)	13 (21.0)	14 (14.1)	0.012
* Staphylococcus aureus*	8 (21.6)	5 (8.1)	13 (13.1)	0.068
* Acinetobacter baumannii*	6 (16.2)	4 (6.5)	10 (10.1)	0.169
* Stenotrophomonas maltophilia*	3 (8.1)	1 (1.6)	4 (4.0)	0.146
* Enterococcus Faecium*	0 (0.0)	2 (3.2)	2 (2.0)	0.527
Others	6 (16.2)	10 (16.1)	16 (16.2)	0.991
Resistance of identified organisms during admission	13 (35.1)	29 (46.8)	42 (42.4)	0.257
Type of resistance				
ESBL	5 (38.5)	19 (65.5)	24 (57.1)	0.101
CRE	5 (38.5)	9 (31.0)	14 (33.3)	0.729
Carbapenem-resistant *Pseudomonas*	2 (15.4)	1 (3.4)	3 (7.1)	0.080
Carbapenem-resistant *Acinetobacter*	3 (23.1)	1 (3.4)	4 (9.5)	0.220
Genes identified				
* bla*_OXA-48_	4 (30.8)	6 (20.7)	10 (23.8)	0.697
* bla*_KPC_	0 (0.0)	1 (3.4)	1 (2.4)	> 0.99

Data are presented as mean ± standard deviation (SD) or *n* (%)

CRE, carbapenem-resistant Enterobacterales; ESBL, extended spectrum β-lactamase; KPC, *Klebsiella pneumoniae* carbapenemase; MDR, multidrug-resistant; OXA, oxacillinase; XDR, extensive drug-resistant

^a^Both positive and negative cultures

Outcomes of patients are listed in Table 4, where the overall rate of superinfection was 8.4% after an average of 15.9 ± 9.9 days from the initiation of antibiotics, with no difference in this rate between the two study groups (6.4% vs. 10.4% in the ADE and continuation group, respectively; *P* = 0.254). The main organisms isolated from these infections were *Klebsiella pneumoniae* (19.2%), *Pseudomonas aeruginosa* (11.5%), and *Acinetobacter baumannii* (11.5%), with no difference in their isolation between the two study groups. A significant difference between the two groups was observed in both mean hospital and ICU length of stay, which were both longer in the ADE group (48.2 ± 88.2 vs. 32.1 ± 43.4 and 19.1 ± 20.0 vs. 14.0 ± 16.5; *P* = 0.016 and 0.002, respectively). Other secondary outcomes (30-day infection recurrence, ICU mortality, and in-hospital mortality) were not significantly different between the two groups.

**Table 4:** Outcomes of patients

Outcome	De-escalation status		Total (*n* = 250)	*P* value
	Yes (*n* = 125)	No (*n* = 125)		
Superinfection	8 (6.4)	13 (10.4)	21 (8.4)	0.254
Days from antibiotic initiation to superinfection	16.1 ± 10.7	15.7 ± 9.9	15.9 ± 9.9	0.856
Superinfection, culture site *n* = 21	*n* = 8	*n* = 13	*n* = 21	
Tracheal aspirate	4 (50.0)	3 (23.1)	7 (33.3)	0.533
Blood	2 (25.0)	4 (30.8)	6 (28.6)	
Urine	1 (12.5)	1 (7.7)	2 (9.5)	
Others	1 (12.5)	5 (38.5)	6 (28.6)	
Superinfection, organisms	*n* = 8	*n* = 13	*n* = 21	0.571
* Klebsiella pneumoniae*	1 (11.1)	4 (23.5)	5 (19.2)	
* Pseudomonas aeruginosa*	1 (11.1)	2 (11.8)	3 (11.5)	
* Acinetobacter baumannii*	2 (22.2)	1 (5.9)	3 (11.5)	
* Candida*	2 (22.2)	1 (5.9)	3 (11.5)	
* Escherichia coli*	0 (0.0)	2 (11.8)	2 (7.7)	
MRSA	1 (11.1)	1 (5.9)	2 (7.7)	
Others	2 (22.2)	6 (35.3)	8 (30.8)	
30-day recurrence of infection after completion of antibiotic therapy *n* = 99	6 (16.2)	7 (11.3)	13 (13.1)	0.545
Require ICU re-admission within 30 days of ICU discharge	21 (16.8)	18 (14.4)	39 (15.6)	0.601
ICU re-admission for Infectious reasons	15 (71.4)	9 (50)	24 (61.5)	0.5
Length of stay				
Length of hospital stay, median (IQR)	26 (14–46)	21 (10–36)	24 (13–41)	0.016
Length of ICU stay	14 (6–23)	8 (4–16)	11 (5–20)	0.002
ICU mortality	42 (33.6)	50 (40.0)	92 (36.8)	0.294
In-hospital mortality	49 (39.2)	57 (45.6)	106 (42.4)	0.306

Data are presented as mean ± standard deviation (SD) or *n* (%); length of stay presented as median and interquartile range (IQR)

ICU, intensive care unit; MRSA, methicillin-resistant *Staphylococcus aureus*

Subgroup analysis of patients in the ADE group whose antibiotics remained de-escalated versus those whose antibiotics were re-escalated is shown in (Supplementary Appendix Table e2). Overall, no difference between the two subgroups was observed, except for age and length of both ICU and hospital stays. The former group tended to be older and stayed longer in the hospital but shorter in the ICU. We conducted a further analysis based on each center (Supplementary Appendix Table e3). We found that de-escalation was seen more in patients admitted to KFHU- K; however, the median (IQR) SOFA score was statistically significantly less in patients admitted to KFHU-K, *P* value = 0.001; that could explain the differences in de-escalation practices. On the other hand, we performed an analysis based on SARS-CoV 2 status. We didn’t find a statistically significant difference between groups with positive and negative screening except for higher total antibiotic therapy in patients with positive SARS- CoV2, *P* = 0.042 (Supplementary appendix Table e4).

## Discussion

Appropriate empirical antibiotic therapy is a major determinant of a patient’s survival, especially for critically ill patients with severe infections. However, there is no gold standard test to diagnose sepsis. Due to this uncertainty in the ICU setting, significant challenges are faced in determining when it is appropriate to de-escalate or discontinue antibiotics [4]. In our study, we observed no difference between the de-escalation and the continuation group in the rate of superinfection, time from broad-spectrum antibiotic initiation to superinfection, rate of 30-day infection recurrence, ICU mortality, and in-hospital mortality. Though, the length of stay was longer in the de-escalation group.

In previous studies, the rate of de-escalation ranged from 10 to 60% in patients with severe sepsis or ventilator-associated pneumonia [8, 13, 16, 23–26]. The definition of de-escalation in these studies was a reduction in the spectrum of administered antibiotics through the discontinuation of antibiotics or switching to an agent with a narrower spectrum. ADE is advised within 24 h of culture finalization [8, 13, 16, 23–26].

Our study found that the antibiotic de-escalation was approximately 7 days. At the same time, de-escalation at 48–72 h of antibiotic initiation was performed only in 18.8% of patients. This delay may be due to the unavailability of rapid diagnostic tests in the microbiology labs of participating centers, in addition to the lack of integration between the automated microbial identification and susceptibility system with the electronic health record system. This could also be an institutional behavior that can be targeted and improved to optimize ADE in the future. In fact, the utilization of advanced technology in terms of using rapid diagnostics with or without clinical decision support systems has resulted in faster time to optimal antibiotic therapy, hence, improved patient outcomes [27]. Moreover, patients in the continuation of therapy group with more positive cultures (49.6% vs. 29.6; *P* value = 0.001) showed a numerically higher resistance (46.8% vs. 35.1%; *P* value = 0.257), which necessitated the need for therapy continuation. The complexity of patients included in the study and the increased resistance observed in the hospital antibiogram might further explain the delayed de-escalation practices.

A previously unblinded randomized non-inferiority trial showed that the ADE group had a higher number of superinfections with the same bacteria and a subsequent increase in the duration of antibiotic therapy compared to the continuation group. Nevertheless, the trial was not powered for this outcome [28]. The overall rate of superinfection in our study was 8.4%, with no difference between both groups (6.4% vs. 10.4%, *P* = 0.254). Additionally, no difference in acquired resistance for superinfections was observed. This is similar to previous findings from other studies, where the effect of ADE on resistance has not been consistent [29–32]. In the crude regression model of our study, patients who had de-escalation had a lower but insignificant risk of superinfection compared with those who did not have de-escalation. Adjustment of the pre-treatment differences with or without treatment differences was not associated with significant differences in the risk of superinfection among those who had de-escalation compared with those who did not have de-escalation (Supplementary Appendix Table e1).

While a decrease in the duration of broad-spectrum antibiotics was observed in the de-escalation group compared to the continuation group (7.2 ± 5.2 vs. 10.3 ± 7.7, *P* < 0.001), no difference was observed between the two groups in the total duration of the antibiotic course. We speculate that the duration of antibiotics may have a more significant impact on resistance rather than de-escalation. A study by Teshome et al. showed that there is a 4% increased risk of new resistance for each additional day of any anti-pseudomonal β-lactam exposure [33]. Another study by Yusuf et al. showed that carbapenem resistance emerged as early as eight days after exposure. The authors recommended de-escalating carbapenem therapy at 48–72 h, supporting an earlier de-escalation time than our study [34].

Notably, the Charlson comorbidity index score was higher in the continuation group than the ADE group (4.8 ± 3.0 vs. 3.7 ± 2.7, *P* = 0.003); hence, the expected hesitancy to de-escalate antibiotics in that group. In the ADE group, patients who were re-escalated demonstrated similar baseline characteristics to those who were not re-escalated. Additionally, no difference was observed in the superinfection rate between the de-escalation then re-escalation group and the de-escalation without the need for a re-escalation group. A potential reason for re-escalation was the deterioration of the patient's status, the same clinical criterion used for initiating antibiotics. Another assumption may have been that the study was not powered enough to detect a difference.

A recent meta-analysis of 20 observational studies demonstrated a lower mortality relative risk of 0.71 with a 95% confidence interval from 0.63 to 0.80 with ADE despite the considerable heterogeneity of the studies [35]. However, according to the unblinded randomized controlled trial by Leone et al., no difference was found in 28-day mortality between the ADE and control groups [28]. Similarly, no difference in ICU mortality (33.6% vs. 40.0%, *P* = 0.294) and in-hospital mortality (39.2% vs. 45.6%, *P* = 0.306) was observed between the ADE group and the continuation group in our study. However, there was a significant difference in hospital stay (48.2 ± 88.2 vs. 32.1 ± 43.4, *P* = 0.016) and ICU length of stay (19.1 ± 20.0 vs. 14.0 ± 16.5, *P* = 0.002). The lack of nursing homes for terminally ill patients in Saudi Arabia and the resultant long hospital stays of patients in tertiary care hospitals could have been a potential confounder.

We believe that this study is one of the few studies to specifically address the superinfection of a different species in the ICU and assess re-escalation after de-escalation in critically ill patients. However, this study has several limitations. First, the delay in ADE may have influenced the lack of difference in outcomes, especially that the total duration was similar in both groups. Second, survivor bias (patients surviving long enough will receive a longer duration of treatment) and bias-by-indication (clinicians selecting not to de-escalate antibiotics for sicker patients) may have been present. Third, the indication of antibiotics cannot be explained by the ICU admission diagnosis reasons or the culture results. Fourth, the de-escalation assessment was complicated, and the decision was mainly based on the clinician assessment, and the possibility of differences in practices for both centers cannot be ruled out. Moreover, details on the reasons for not performing ADE were not collected due to the retrospective nature of the study. Fifth, the reasons for the re-escalation of antibiotics after de-escalation were also not collected due to the study's retrospective nature and the lack of appropriate, pertinent documentation in the electronic health record system. Our results generally demonstrate the safety of antibiotic de-escalation, as there was no statistical difference in superinfection or mortality between both groups. However, the delay in de-escalation practices could affect the generalizability of our findings. Other potential benefits that were not explicitly assessed in our study include a lower risk of antibiotic-related adverse effects, including *Clostridioides difficile* infection, and potential cost savings as ADE allows a reduction in the duration of expensive antibiotics and subsequent use of less expensive antibiotics for treatment continuation [36, 37].

## Conclusion

Our study found no significant differences in superinfection rate or mortality among critically ill patients whose broad-spectrum antibiotics were de-escalated compared with patients in the continuation of therapy group. Future studies determining the impact of rapid diagnostics on ADE in a high resistance rate setting are warranted.

## Supplementary Information

Below is the link to the electronic supplementary material.

Supplementary file 1 (DOCX 260 KB)

## Data Availability

All data are available upon request.
