# Supplementary material for: Effect of Broad-Spectrum Antibiotic De-escalation on Critically Ill Patient Outcomes: A Retrospective Cohort Study
Source: J Epidemiol Glob Health. 2023 Jun 9;13(3):444–52. doi: 10.1007/s44197-023-00124-1 (PMC10255942; doi:10.1007/s44197-023-00124-1)
Supplement: Supplementary file 1 — (DOCX 260 KB) [file 44197_2023_124_MOESM1_ESM.docx]

**Supplementary Appendix**

**Effect of broad-spectrum antibiotic de-escalation on critically ill patient outcomes: A retrospective cohort study**

**Figure e1: Guidance for the Decision on the Spectrum of Antibiotics**

**Figure e2:** Patient Screening and Selection

**Figure e3.** Antibiotic de-escalation and re-escalation status among included ICU patients

**Table e1:** Univariate and multivariate logistic regression analysis for potential predictors of Superinfection by de-escalation status (N=250)

|  | Odds ratio | 95% confidence | | P-value |
| --- | --- | --- | --- | --- |
|  |  | Lower | Upper |  |
| **Crude model** | 0.59 | 0.24 | 1.48 | 0.259 |
| **Model-1 adjusted for pre-treatment differences*** | 0.99 | 0.36 | 2.77 | 0.987 |
| **Model-2 adjusted for pre-treatment and treatment differences**** | 2.27 | 0.53 | 9.81 | 0.272 |

* Adjusted for age, admission diagnosis, Charlson comorbidity index score, APACHE score, and antibiotics used before ICU admission, including Meropenem and Piperacillin/tazobactam.

** Adjusted for the variables in model-1 in addition to the duration of primary broad-spectrum antibiotics, discontinuation of broad-spectrum antibiotics in 48-72 hours, concurrent use of antibiotics, and positive culture, including Escherichia coli and Coagulase-negative staphylococci.

**Table e 2.** Baseline characteristics and outcome of patients whose broad-spectrum antibiotics de-escalated (n=125)

| **Characteristic** | **De-escalation without the need for re-escalation** | **De-escalation then re-escalation** | ***P* value** |
| --- | --- | --- | --- |
|  | **N=68** | **N=57** |  |
| **Age, mean± SD** | 63.5±20.2 | 56.5±21.4 | 0.049 |
| **Gender (male), n (%)** | 41 (60.3) | 42 (73.7) | 0.114 |
| **BMI, mean± SD** | 28.3±7.6 | 27.9±6.7 | 0.818 |
| **Invasive ventilation, n(%)** | 37 (54.4) | 39 (68.4) | 0.110 |
| **Charlson Comorbidity index, mean± SD** | 4.1±2.8 | 3.2±2.4 | 0.061 |
| **APACHE score, mean± SD** | 18.7±6.6 | 20.6±9.1 | 0.339 |
| **SOFA score, mean± SD** | 7.3±3.5 | 7.9±3.4 | 0.616 |
| **History of MDRs or XDR 12 months before ICU admission, n(%)** | 7 (10.3) | 7 (12.3) | 0.726 |
| **Cultures positive, n(%)** | 19 (27.9) | 18 (31.6) | 0.657 |
| **Any resistance, n(%)** | 6 (31.6) | 7 (38.9) | 0.642 |
| **Resistance type, n(%)** | | | |
| ESBL | 1 (16.7) | 4 (57.1) | 0.266 |
| CRE | 3 (50.0) | 2 (28.6) | 0.592 |
| **Carbapenem-resistant *Acinetobacter*** | 1 (16.7) | 1 (14.3) | >0.99 |
| **Carbapenem-resistant *Pseudomonas*** | 2 (33.3) | 1 (14.3) | 0.559 |
| **Genes identified, n(%)** | 2 (33.3) | 2 (28.6) | >0.99 |
| **Genes identified, n(%)** | | | |
| *bla*_IMI_ | 0 (0.0) | 0 (0.0) | NA |
| *bla*_OXA-48_ | 2 (33.3) | 2 (28.6) | >0.99 |
| *bla*_NDM_ | 0 (0.0) | 0 (0.0) | NA |
| *bla*_KPC_ | 0 (0.0) | 0 (0.0) | NA |
| *bla*_VIM_ | 0 (0.0) | 0 (0.0) | NA |
| **Duration of a primary broad-spectrum antibiotic (days), mean± SD** | 8.1±5.7 | 6.1±4.3 | .010 |
| **The total duration of the antibiotic course (including broad-spectrum and de-escalated antibiotic), mean± SD** | 11.4±8.1 | 13.6±8.5 | .071 |
| **Superinfection, n(%)** | 2 (2.9) | 6 (10.5) | 0.140 |
| **Length of hospital stay, mean± SD** | 53.4±112.1 | 42.0±46.4 | 0.049 |
| **Length of ICU stay, mean± SD** | 15.9±19.1 | 23.1±20.6 | 0.010 |
| **Days from hospital admission to ICU admission, mean± SD** | 3.8±9.9 | 2.5±4.7 | 0.932 |

APACHE, Acute Physiology and Chronic Health Evaluation; CKD, chronic kidney disease; COPD, chronic obstructive pulmonary disease; CRE, carbapenem-resistant Enterobacterales; CVA, cerebrovascular accident; ESBL, extended-spectrum β-lactamase; ICU, intensive care unit; KPC, *Klebsiella pneumoniae* carbapenemase; MDR, multidrug-resistant; OXA, oxacillinase; SD, standard deviation; SOFA, Sequential Organ Failure Assessment; TIA, transient ischemic attack; XDR, extensive drug-resistant

**Table e3:** Antibiotic De- escalation & outcome per center

|  | **KFSHRC-J**  **N=121** | **KFHU-K**  **N=129** | **Total**  **N=250** | **p-value** |
| --- | --- | --- | --- | --- |
| **Age (years), mean± SD** | 60.0±17.8 | 62.1±21.3 | 61.1±19.7 | 0.407 |
| **Gender, male, n(%)** | 68 (56.2%) | 85 (65.9%) | 153 (61.2%) | 0.116 |
| **APACHE score, median (IQR)** | 20 (16-26) | 19 (14-26) | 19 (15-26) | 0.218 |
| **SOFA score, mean± SD** | 8 (6-11) | 5 (3-8) | 8 (5-10) | 0.001 |
| **Charlson Comorbidity Index, median (IQR)** | 4 (2-7) | 4 (1-6) | 4 (2-6) | 0.166 |
| **Invasive ventilation, n(%)** | 68 (56.2%) | 78 (60.5%) | 146 (58.4%) | 0.49 |
| **De-escalation status, n(%)** |  |  |  |  |
| **De-escalation of antibiotics, n(%)** | 36 (29.8%) | 89 (69.0%) | 125 (50.0%) | <0.001 |
| **History of MDRs or XDR 12 months prior to ICU admission** | 20 (16.5%) | 9 (7.0%) | 29 (11.6%) | 0.018 |
| **Superinfection, n(%)** | 12 (9.9%) | 9 (7.0%) | 21 (8.4%) | 0.040 |
| **Duration of primary broad-spectrum antibiotic days, median (IQR)** | 7 (5-12) | 7 (4-11) | 7 (4-11) | 0.299 |
| **The total duration of the Antibiotic course, median (IQR)** | 9 (6-14) | 11 (7-17) | 10 (6-16) | 0.121 |
| **Length of hospital stay, median (IQR)** | 24 (12-46) | 24 (13-37) | 24 (13-41) | 0.598 |
| **Length of ICU stay, median (IQR)** | 9 (4-18) | 11 (5-22) | 11 (5-20) | 0.108 |
| **Days between Hospital admission to ICU admission, n median (IQR)** | 1 (0-5) | 1 (0-3) | 1 (0-3) | 0.263 |

King Faisal Specialist Hospital in Jeddah (KFSHRC-J); King Fahad Hospital of the University (KFHU); APACHE, Acute Physiology, and Chronic Health Evaluation; SD, standard deviation; SOFA, Sequential Organ Failure Assessment; ICU, intensive care unit; IQR, interquartile range; MDR, multidrug-resistant; XDR, extensive drug-resistant

**Table e4:** Antibiotic De-escalation & outcome per SARS‑CoV‑2 Status

|  | **SARS CoV 2**  **Positive**  **N=40** | **SARS CoV 2 Negative**  **N=210** | **Total**  **N=250** | **p-value** |
| --- | --- | --- | --- | --- |
| **Age (years), mean± SD** | 66.0±19.1 | 60.1±19.7 | 61.1±19.7 | 0.085 |
| **Gender (male), n(%)** | 26 (65.0) | 127 (60.5% | 153 (61.2) | 0.590 |
| **APACHE score, median (IQR)** | 20 (14-29) | 19 (15-25) | 19 (15-26) | 0.762 |
| **SOFA score, median (IQR)** | 4 (3-5) | 8 (5-10) | 8 (5-10) | 0.046 |
| **Charlson Comorbidity Index, median (IQR)** | 4 (2-6) | 4 (2-6) | 4 (2-6) | 0.789 |
| **Invasive ventilation, n(%)** | 17 (42.5) | 129 (61.4) | 146 (58.4) | 0.026 |
| **De-escalation status, n(%)** | 16 (40.0) | 109 (51.9) | 125 (50.0) | 0.168 |
| **History of MDRs or XDRs 12 months prior to ICU admission, n(%)** | 3 (7.5) | 26 (12.4) | 29 (11.6) | 0.589 |
| **Superinfection, n(%)** | 3 (7.5) | 18 (8.6) | 21 (8.4) | >0.99 |
| **Duration of primary broad-spectrum antibiotic, days, median (IQR)** | 8 (6-14) | 7 (4-11) | 7 (4-11) | 0.042 |
| **The total duration of the Antibiotic course, days, median (IQR)** | 11 (7-19) | 10 (6-15) | 10 (6-16) | 0.182 |
| **Length of hospital stay, median (IQR)** | 23 (10-39) | 24 (14-41) | 24 (13-41) | 0.576 |
| **Length of ICU stay, median (IQR)** | 14 (6-32) | 10 (5-19) | 11 (5-20) | 0.077 |
| **Days between Hospital admission to ICU admission, median (IQR)** | 0 (0-2) | 1 (0-4) | 1 (0-3) | 0.140 |

SARS CoV 2, Severe acute respiratory syndrome coronavirus 2; APACHE, Acute Physiology, and Chronic Health Evaluation; SD, standard deviation; SOFA, Sequential Organ Failure Assessment; ICU, intensive care unit; IQR, interquartile range; MDR, multidrug-resistant; XDR, extensive drug-resistant
